# Supplementary material for: DNA methylation profiling to determine the primary sites of metastatic cancers using formalin-fixed paraffin-embedded tissues
Source: Nat Commun. 2023 Sep 14;14:5686. doi: 10.1038/s41467-023-41015-0 (PMC10502058; doi:10.1038/s41467-023-41015-0)
Supplement: Supplementary file 1 — Supplementary Information [file 41467_2023_41015_MOESM1_ESM.pdf]

DNA methylation profiling to determine the primary sites of metastatic cancers using formalin-fixed paraffin-embedded tissues

Supplementary Methods

Patient Selection Protocols

Patients enrolled in this study were selected from the authors' hospitals. For the retrospective primary cancer cohort, patients were diagnosed with one of ten common cancers after standard procedures, including medical history, physical examination, blood tests, imaging studies, hematoxylin and eosin (H&E) staining, and immunohistochemistry (IHC) analysis of resected or biopsied tissue. The tumor cell content of each patient's sample should be at least 70%. Patients treated with chemotherapy, radiotherapy, or other therapies were excluded. Patients with more than one primary tumor site were also excluded. Ultimately, we selected fresh frozen tumor tissue from 498 patients for the training cohort.

Similarly, the cohort of patients with metastatic cancer was diagnosed according to standard assessment. Board-certified pathologists reviewed the results of histologic or cytologic examinations. FFPE samples with less than 10% tumor cells were not eligible for testing, and sufficient FFPE tissue was required for DNA methylation profiling. Notably, sequencing results from 40 patients were excluded from further analysis due to poor library quality, including low DNA input, low bisulfite convention rates, or insufficient sequencing reads. Of the remaining 223 patients, 215 were included in the validation cohort. FFPE samples from the remaining eight patients were used for library evaluation.

The prospective cohort included 68 patients with CUP diagnosed according to the US National Comprehensive Cancer Network (NCCN) recommendations [1]. Patients' primary sites were clarified by further clinical evaluation, IHC analysis, or both. All 68 patients were included in the study.

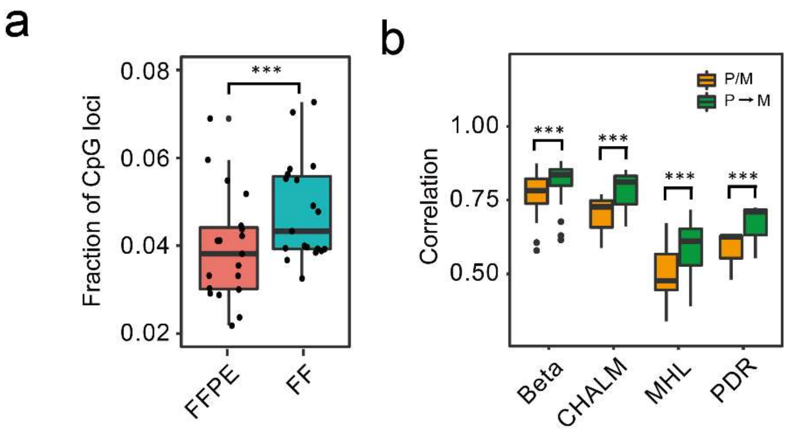

Supplementary Fig. 1. Comparison of RRBS data from different sample types. (a) Box plots show the fraction of CpGs detected in the two library types. The percentages of CpGs detected at ≥5x coverage were computed by randomly sampling 800K sequencing reads from 19 paired FFPE-RRBS (red rectangle) and FF-RRBS (blue rectangle). The calculation is based on a total number of 2,8217,448 CpGs in the human genome (the UCSC version hg19; GRCh37.p13). In the boxplot, the center line, box limits and whiskers separately indicate the median, upper and lower quartiles and 1.5x interquartile range. *** indicates a significant difference at $P<0.001$, determined by two-sided Wilcoxon rank sum test. **(b)** Correlations of DNA methylation profiles between metastatic tissues and the corresponding primary tumor tissues (P→M; green rectangle) or the primary tumors that originated at the metastatic site (P/M; yellow rectangle). Of the total 251 metastatic samples, 36 were obtained by biopsy from three different tissues, namely liver (n = 25), lung (n = 8), and stomach (n = 3). For these tissues, corresponding primary tumor samples were available for analysis (57 lung, 48 stomach, and 46 liver). The correlation between each selected metastatic sample and two types of primary tumor samples, namely those corresponding to the primary tumor (n=405) and the primary tumor originating from the metastatic site (n=151), was calculated and then compared. CGI methylation was assessed using the following four methods, including beta value, CHALM, MHL, and PDR, as described in the Methods section. Primary cancer methylation profiles were derived from the FF-RRBS libraries in the training dataset. In the boxplot, the center line, box limits and whiskers separately indicate the median, upper and lower quartiles and 1.5x interquartile range. *** indicates a significant difference at $P<0.001$, determined by two-sided Wilcoxon rank sum test. Source data are provided as a Source Data file.

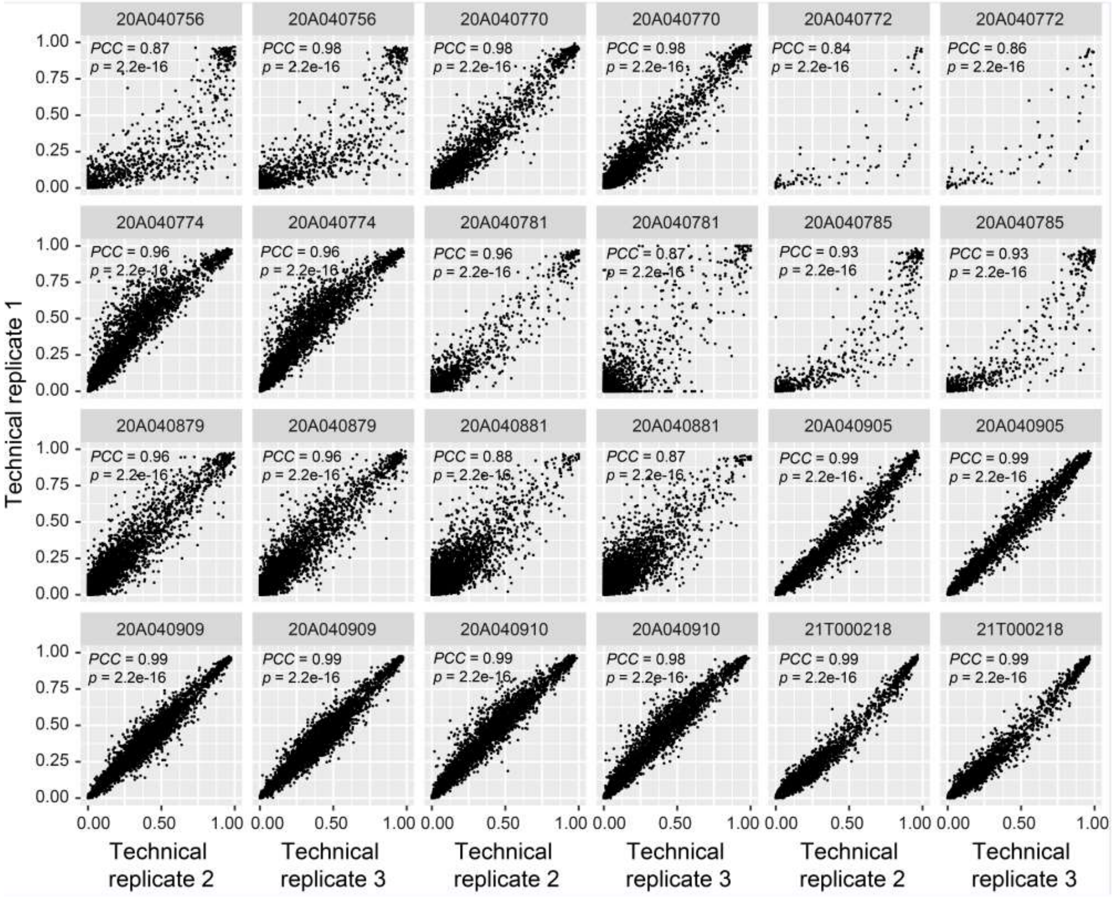

Supplementary Fig. 2. Comparison of beta value between triplicates of selected FFPE RRBS libraries. Each point represents a CGI, and the horizontal and vertical coordinates represent the beta value of the corresponding CGI in different technical replicates, respectively. The sample ID is listed in the gray box. PCC: Pearson correlation coefficient; p: *P* value, determined by two-sided Student's *t*-test. Source data are available in a supplementary file.

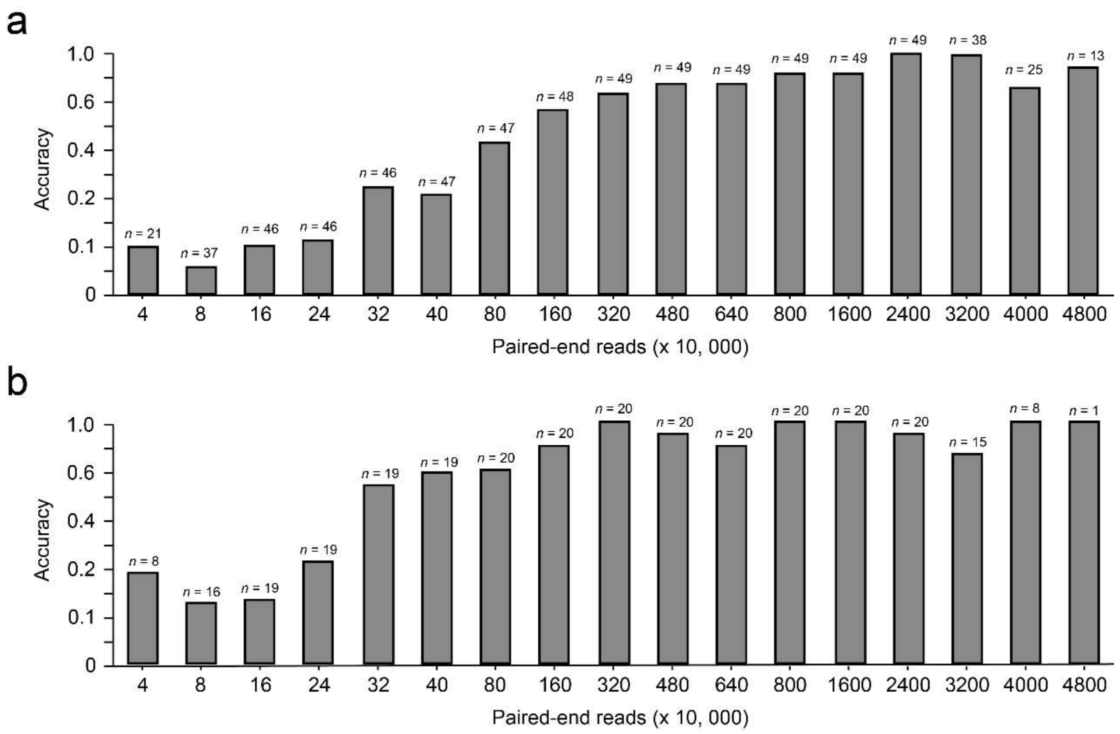

Supplementary Fig. 3. Prediction accuracies were calculated for different inputs of sequencing data in the low (a) and high (b) tumor content groups. FFPE-RRBS samples were divided into low (≤ 0.5) and high tumor content (> 0.5) groups. The top 49 low tumor content or 20 high tumor content FFPE-RRBS libraries with more than 24 million paired-end reads were subjected to downsampling analysis. After randomly dropping a fraction of the sequencing reads, the remaining data was used to test the prediction accuracy of BELIVE. Source data are available in a supplementary file.

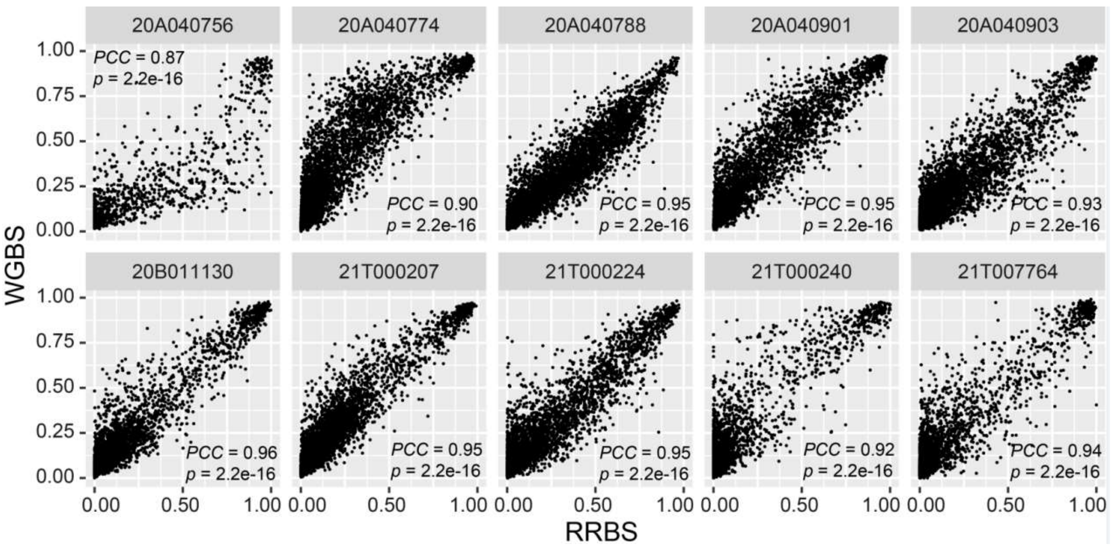

Supplementary Fig. 4. Comparison of beta value between RRBS and WGBS library of selected FFPE samples. Each point represents a CGI, and the horizontal and vertical coordinates represent the beta value of the corresponding CGI in RRBS and WGBS libraries, respectively. The sample ID is listed in the gray box. PCC: Pearson correlation coefficient; p: *P* value, determined by two-sided Student's *t*-test. Source data are available in a supplementary file.

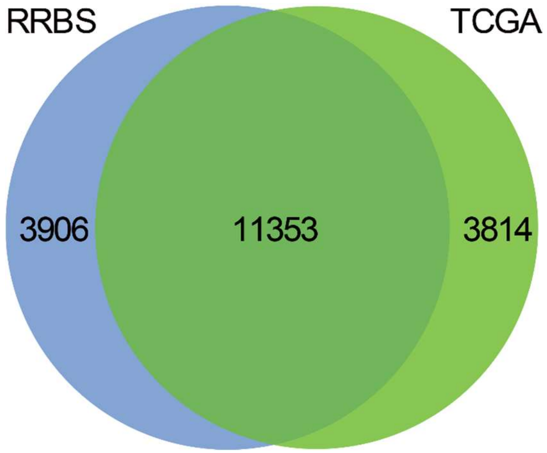

Supplementary Fig. 5. Comparison of covered CGIs by RRBS and the Illumina 450K DNA methylation microarray. Approximately 60% CGIs (n=11,353) were shared by both, while 3,906 CGIs (20%) were specific to the RRBS dataset and 3,814 CGIs (20%) were unique to the Illumina 450K DNA methylation microarray dataset. Source data are available in a supplementary file.

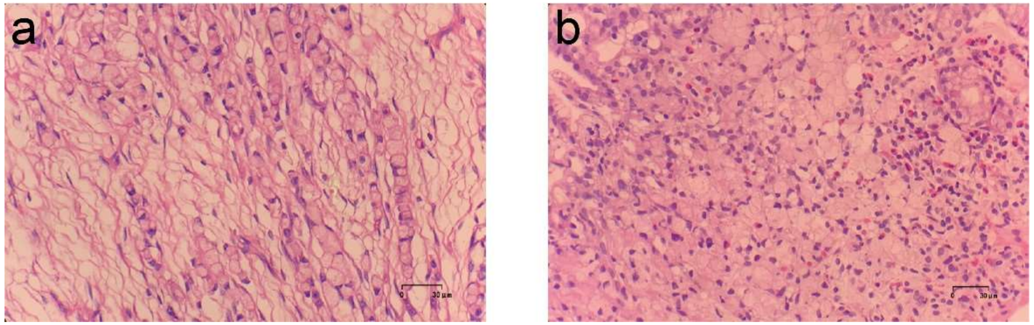

Supplementary Fig. 6 Hematoxylin and eosin (H&E) staining on biopsied tissue from inguinal lymph nodes (**a**) and gastric lesions (**b**) of the CUP patient (200x). Assays were repeated on a series of sections (n=3) and images were evaluated by independent pathologists. The images shown were representative of the series. The scale bar (30 μm) is shown in the lower right corner (a and b). Source data are available in a supplementary file.

Supplementary Table 1. Comparison of the sequencing results of paired fresh frozen and FFPE samples

	Frozen		FFPE		
**Reads**	**Mean (95% CI)**	**Range**	**Mean (95% CI)**	**Range**	**Unadjusted *P* value**
Total reads (in millions)	37.42 (31.2-43.64)	12.94-64.27	40.61 (29.47-51.75)	8.93-92.85	0.63
Mapped reads (%)	68.96 (67.75-70.17)	62.82-73.46	66.47 (64.26-68.68)	55.57-71.42	0.06
Conversion ratio (%)	99.88 (99.87-99.89)	99.8-99.91	99.63 (99.57-99.7)	99.23-99.82	0.000000216
Mapped on-CGI reads (%)	18.92 (17.67-20.16)	14.5-25.4	28.51 (25.7-31.32)	15.98-40.49	0.00000222
Mean insert size (bp)	117.91 (113.73-122.09)	108.06-141.02	82.38 (76.31-88.45)	60.01-108.93	1E-10

*： *P* value was calculated using two-sided Wilcoxon rank sum test.

Supplementary Table 2. Comparison of the sequencing results of paired primary cancer and metastatic cancer samples

	Primary		Metastatic		
**Reads**	**Mean (95% CI)**	**Range**	**Mean (95% CI)**	**Range**	**Unadjusted *P* value***
Total reads (in millions)	59.8 (55.66-63.94)	52.32-81.21	59.41 (53.05-65.77)	28.21-73.72	0.949635367
Mapped reads (%)	69.03 (68.01-70.05)	65.15-71.78	68.35 (67.43-69.27)	65.39-71.89	0.541976886
Conversion ratio (%)	99.64 (99.62-99.66)	99.53-99.71	99.6 (99.57-99.62)	99.52-99.67	0.161102236
Mapped on-CGI reads (%)	32.03 (29.75-34.3)	24.39-37.56	33.75 (31.7-35.8)	25.92-39.03	0.484912213
Mean insert size (bp)	81.82 (79.85-83.79)	74.94-88.8	79.87 (73.5-86.24)	71.55-88.69	0.102958017

*: *P* value was calculated using two-sided Wilcoxon rank sum test

Supplementary Table 3. Predictive AUC of seven machine learning classifiers trained on four methylation evaluation methods

Methods	Beta	CHALM	MHL	PDR
Adaboost	0.88	0.56	0.88	0.52
KNN	0.81	0.58	0.80	0.54
LGR	0.95	0.58	0.92	0.60
LinearSVC	0.95	0.54	0.92	0.57
NB	0.58	0.53	0.55	0.54
RF	0.88	0.57	0.86	0.58
SVM	0.90	0.61	0.85	0.59

Supplementary Table 4. Performance of BELIVE in predicting the primary sites of metastatic cancer

Primary Origin	Precision	Recall	F1-score	AUC-ROC	Count
Thyroid	0.87	0.87	0.87	0.99	31
Colon and rectum	1.00	0.90	0.95	0.98	31
Stomach	0.80	0.69	0.74	0.91	29
Lung	0.96	0.88	0.92	0.97	26
Breast	0.96	0.85	0.90	0.99	26
Head and neck	0.52	0.80	0.63	0.9	20
Ovarian	0.83	0.88	0.86	0.92	17
Esophagus	0.63	0.59	0.61	0.84	17
Liver and gallbladder	0.67	0.89	0.76	0.89	9
Cervix	0.86	0.67	0.75	0.97	9

Supplementary Table 5. Comparison of the predicted primary sites based on paired RRBS and WGBS

Sample ID	Diagnosed cancer type	TOP 1 (RRBS)	TOP 1 probability (RRBS)	TOP 1 (WGBS)	TOP 1 probability (WGBS)
20A040756	Breast cancer	Breast cancer	0.873631649	Breast cancer	0.887141453
20A040774	Breast cancer	Breast cancer	0.892821993	Breast cancer	0.866749984
21T000207	Lung cancer	Lung cancer	0.899592935	Lung cancer	0.880496914
20B011130	Thyroid cancer	Thyroid cancer	0.896693165	Thyroid cancer	0.886394896
20A040903	Colon and rectum cancer	Colon and rectum cancer	0.901050526	Colon and rectum cancer	0.884804459
20A040901	Colon and rectum cancer	Colon and rectum cancer	0.90100303	Colon and rectum cancer	0.892934189
21T000240	Ovarian cancer	Ovarian cancer	0.897056336	Ovarian cancer	0.89206101
21T007764	Ovarian cancer	Ovarian cancer	0.892808062	Ovarian cancer	0.892052615
21T000224	Esophagus cancer	Esophagus cancer	0.883434625	Esophagus cancer	0.893459593
20A040788	Stomach cancer	Stomach cancer	0.91052172	Stomach cancer	0.895398931

Supplementary Table 6. BELIVE performance on the Illumina 450K methylation microarray dataset

Primary Origin	Precision	Recall	F1-score	AUC-ROC	Count
Lung	0.99	0.81	0.89	0.98	828
Colon and rectum	0.91	0.97	0.94	1	786
Breast	0.99	0.98	0.99	1	783
Head and neck	0.91	0.96	0.94	1	528
Thyroid	1.00	0.83	0.91	0.99	503
Stomach	0.64	0.99	0.78	0.99	395
Liver and gallbladder	0.95	1.00	0.97	1	377
Cervix	0.97	0.88	0.92	0.99	307
Esophagus	0.99	0.82	0.90	0.92	185
Ovarian	0.77	1.00	0.87	1	10

Supplementary References

1. Ettinger, D.S., et al., *NCCN Clinical Practice Guidelines Occult primary.* J Natl Compr Canc Netw, 2011. **9**(12): p. 1358-95.
