# Supplementary material for: DNA methylation profiling to determine the primary sites of metastatic cancers using formalin-fixed paraffin-embedded tissues
Source: Nat Commun. 2023 Sep 14;14:5686. doi: 10.1038/s41467-023-41015-0 (PMC10502058; doi:10.1038/s41467-023-41015-0)
Supplement: Supplementary file 4 — Description of Additional Supplementary Files [file 41467_2023_41015_MOESM4_ESM.pdf]

Description of Additional Supplementary Files

Supplementary File 1
Description:
Supplementary Data 1 Characteristics of patients with primary and metastatic cancer

Supplementary File 2
Description:
Supplementary Data 2. Summary of quality control of the RRBS data

Supplementary File 3
Description:
Supplementary Data 3. Comparison of primary sites predicted by BELIVE and clinicopathologic analysis

Supplementary File 4
Description:
Supplementary Data 4. Summary of antibodies used in IHC assays

Supplementary File 5
Description: Supplementary information

Supplementary File 6
Description: Source Data
